# A Role for Epigenetic Regulation in the Adaptation and Stress Responses of Non-model Plants

**DOI:** 10.3389/fpls.2019.00246

**Published:** 2019-03-01

**Authors:** Flávia Thiebaut, Adriana Silva Hemerly, Paulo Cavalcanti Gomes Ferreira

**Affiliations:** Laboratório de Biologia Molecular de Plantas, Instituto de Bioquímica Médica Leopoldo de Meis, Universidade Federal do Rio de Janeiro, Rio de Janeiro, Brazil

**Keywords:** DNA methylation, histone modification, epigenetic variation, stress, environment

## Abstract

In recent years enormous progress has been made in understanding the role of epigenetic regulation response to environmental stimuli, especially in response to stresses. Molecular mechanisms involved in chromatin dynamics and silencing have been explained, leading to an appreciation of how new phenotypes can be generated quickly in response to environmental modifications. In some cases, it has also been shown that epigenetic modifications can be stably transmitted to the next generations. Despite this, the vast majority of studies have been carried out with model plants, particularly with Arabidopsis, and very little is known on how native plants in their natural habitat react to changes in their environment. Climate change has been affecting, sometimes drastically, the conditions of numerous ecosystems around the world, forcing populations of native species to adapt quickly. Although part of the adaptation can be explained by the preexisting genetic variation in the populations, recent studies have shown that new stable phenotypes can be generated through epigenetic modifications in few generations, contributing to the stability and survival of the plants in their natural habitat. Here, we review the recent data that suggest that epigenetic variation can help natural populations to cope to with change in their environments.

## Introduction of Epigenetic Regulation

Plants are sessile organisms that are exposed to different environmental conditions. Consequently, plants developed sophisticate mechanisms of gene regulation to ensure the survival upon environmental fluctuations. Plants sense the signals from the environment and transmitted them through a cascade of signal transduction, triggering the accumulation of transcription factors that activate gene expression that can result in adaptation to environmental challenges (Mirouze and Paszkowski, 2011). Another important mechanism of gene regulation in response to stresses is epigenetic regulation, which consists of covalent modifications of DNA and histones, affecting transcriptional activity of chromatin without changing DNA sequence (Iwasaki and Paszkowski, 2014). Chromatin structure is composed of nucleosomes formed by the interaction of histone proteins with DNA, allowing packaging of the DNA in the nucleus (Alberts et al., 2002). Because gene expression is dependent of access to DNA, thus the level of condensation of chromatin is important to this regulation. Euchromatin can be associated with transcriptional active regions, while heterochromatin is normally a transcriptional silenced region, with hypermethylation of DNA and specific modification of histones (Vaillant and Paszkowski, 2007). Studies have highlighted three epigenetic marks: DNA methylation, histone modifications and small RNAs. Important, in many cases small RNAs can trigger DNA methylation and chromatin modification (Meyer, 2015).

In plants, epigenetic modification by DNA methylation has been thoroughly studied and the mechanisms controlling DNA methylation inheritance is well established (Martienssen and Colot, 2001; Takeda and Paszkowski, 2006). DNA methylation consists mostly in adding a methyl group at the fifth carbon position of a cytosine ring, and, different to what happens in animals, plants have three sites that frequently can suffer methylation: CG, CHG (where H is A, C, or T), and CHH (Law and Jacobsen, 2010). Studies revealed that different enzymes are responsible for methylation in each contexts: MET1 DNA methyltransferase maintains the CG methylation, methyltransferase CHROMOMETHYLASE3 – CMT3 maintains the CHG methylation and DOMAINS REARRANGED METHYLTRANSFERASE – DRM1/DRM2 or CMT2 methyltransferase are responsible for CHH methylation (Ronemus et al., 1996; Chan et al., 2006; Du et al., 2012). In addition, short interfering RNAs (siRNAs) can guide RNA-directed DNA Methylation (RdDM) pathway. In the nucleus, siRNAs are derived from long dsRNAs transcription by RNA Polymerase IV and processed by DICER-LIKE 3 (DCL3). Next, siRNAs are formed and exported to the cytoplasm to be incorporated into the RISC complex containing ARGONAUTE 4 (AGO4). Then, siRNA-AGO4 is transported to the nucleus, where siRNA align with their target, a nascent scaffold transcript from RNA Polymerase V, and recruit DNA methyltransferase to silencing its target (Matzke and Mosher, 2014). Transposons silencing can be due the DNA methylation resulting in a protection of genome integrity (Chomet et al., 1987; Ito, 2013). In addition, DNA methylation is also occurring in gene-coding regions affecting gene expression. Curiously, in Arabidopsis, one-third of methylated genes occur in transcribed regions, and 5% of genes showed methylation in promoter regions, suggesting that many of these are epigenetically regulated by DNA methylation (Zhang et al., 2006).

Modification of DNA methylation profiles in plant can cause phenotypic variation. For instance, demethylation of rice genomic DNA cause an altered pattern of gene expression, inducing dwarf plants (Sano et al., 1990). A 16% reduction in the 5-methylcitosine (m^5^C) content was observed in rice plants treated with DNA demethylating agents, and this reduction in DNA methylation leads to phenotypic changes observed in the progeny. According to the above mentioned, stress can also result in changes in DNA methylation. DNA methylation content can also be regulated in response to abiotic stress (Dowen et al., 2012). Experiments in maize and Arabidopsis showed that cold stress might induce modification of the DNA methylation status (Steward et al., 2002; Song et al., 2012). Vernalization treatments result in reduction of levels of DNA methylation and induced the initiation of flowering (Burn et al., 1993). Some stress-induced modifications are reversed to the basal level; however, some of these modifications may be stable and heritable, being named the epigenetic “stress memory” (Kinoshita and Seki, 2014). The knowledge of these stress memories can increase our understanding the processes of plant adaptation to stresses.

**FIGURE 1:**
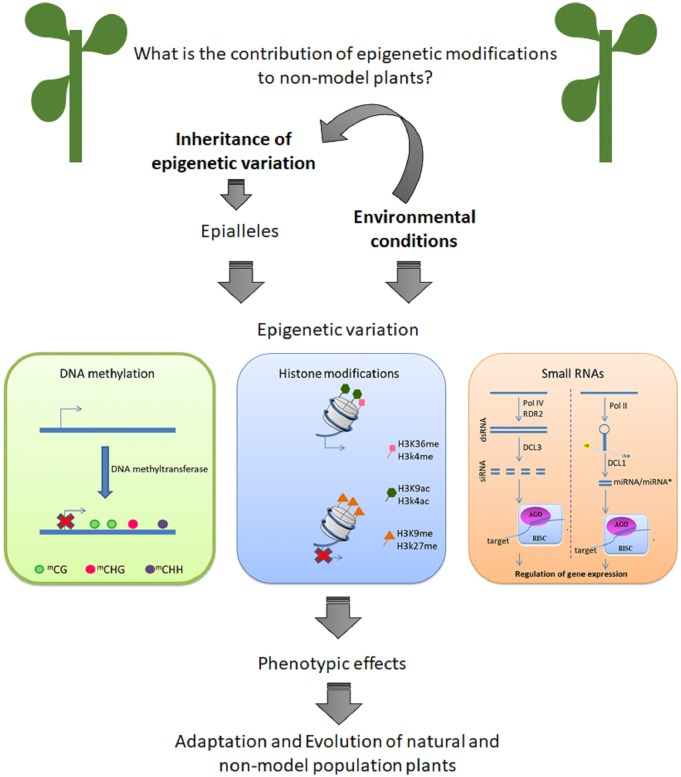
Epigenetic variation can contribute to adaptation and evolution of non-model plants.

An important question is: what is the contribution of epigenetic modification to phenotypic variation in native plants in their environment? Here, we review recent data that suggest that epigenetic variation can contribute to natural populations to cope to with changes in their environments (Figure 1). Is important to know that epigenetic can be define as mitotically and/or meiotically heritable variation in phenotype (Niederhuth and Schmitz, 2014). Despite of the importance of other mechanisms of epigenetic modification, DNA methylation is the better studied process in non-model plants. Firstly, we describe epigenetic changes as heritable characteristics. Next, we discuss recent studies performed with non-model plants. Is important to highlight that the knowledge of epigenetic mechanisms from model species is useful in non-model systems, suggesting gene regulation and the components of epigenetic machinery (Richards et al., 2017). However, non-model plants are becoming very attractive study material due their ability to adapt to extreme environments.

## Inheritance of Epigenetic Variation

Epigenetic marks, such as DNA methylation, can be modified and result in an epigenetic response. Transgenerational epigenetic inheritance requires that epigenetic marks can be transmitted to the progeny (Hauser et al., 2011). Thus, we can say that epigenetic marks might be transmitted through mitosis and sometimes also meiosis. The variation in methylation of the same gene between different plants is denominated epialleles. Epialleles differ in the number or distribution of methylated nucleotides at specific gene sequences and it is important to known that different epialleles can result in different phenotypes which are heritable in a new generation. In maize, it has been described the involvement of transposable elements (TE) regulation during plant development and the impact in the inheritance of epialleles (Martienssen et al., 1990). Moreover, a naturally occurring mutant of *Linaria vulgaris* is an example that suggests a transgenerational epigenetic inheritance (Cubas et al., 1999). Authors showed that the levels of DNA methylation of the CYCLOIDEA cause phenotypic alterations in flower symmetry and these are maintained for hundreds of years. Is important to highlight that some epigenetic marks may result in heritable phenotypic variation whereas others are not (Baulcombe and Dean, 2014). Until now, research carried out has not fully explained the mechanisms involved, but data show that DNA methylation is an epigenetic mark easier to pass through generations. In plants some germline cells are descended from somatic cells and they carry epigenetic marks, which can contribute with the heritability of epigenetic marks.

The majority of studies on epigenetic inheritance focused on DNA methylation (Kalisz and Purugganan, 2004). To understand the mechanisms involved in transgenerational epigenetic inheritance is necessary to picture out how of epigenetic marks are propagation during gametophyte development is carried out, which develops through mitotic divisions from the meiotic products. In Arabidopsis, epigenetic marks are lost in the somatic cells of pollen to activate the transposons, but this RNA can be server as precursor of siRNA production that can silenced this transposon in germ cells and give rise to the next generation (Slotkin and Martienssen, 2007). Thus, studies suggest that heritable epigenetic marks may result in heritable phenotypic variation, influencing fitness, and so be subject to natural selection (Baulcombe and Dean, 2014). Unlike mammals, CG and CHG DNA methylation were kept in three haploid cell types from developing pollen (Calarco et al., 2012). Despite the loss of CHH methylation in retrotransposons in microspores and sperm cells, the action of siRNAs with 24 nucleotides in length can restore methylation by *de novo* DNA methyltransferase activity. This result showed the importance of small RNAs (sRNA) in the methylation process. Moreover, DNA methylation via sRNA is also involved in regulation of TEs and repeats, whose reduction in DNA methylation can result in increased movement of TEs and can also influence genetic variation (Matzke and Mosher, 2014). As replication of methylated DNA sequences results in hemimethylation, where only one strand of the DNA double helix is methylated, plants have a METHYLTRANSFERASE1 (MET1) that is involved in replication of CG methylation and consequently the hemimethylated DNA can server as copy to newly synthesized strand. Interestingly, a study of a mutant for MET1 revealed that the maintenance of methylation in somatic tissues was lost during gametogenesis (Saze et al., 2003).

Is important to recognize that plants can sense the environmental conditions during vegetative growth and this could result in epigenetic modifications in a cell lineage that can generate a germline (Mirouze and Paszkowski, 2011). Studies using the model plant Arabidopsis have showed that stress-induced transgenerational responses depend on changes in DNA methylation (Boyko et al., 2010; Lang-Mladek et al., 2010). Based on this observation, it is possible that phenotypic effects caused by epialleles are inherited across generations and influenced by environmental conditions also in native plants. Therefore, heritable epialleles will influence plant evolution through their effects on both phenotypic trait distributions and fitness. In addition, many plants are propagated asexually through clonal reproduction, where meiotic epigenetic reset does not occur. The epigenetic information among clonal generations is more effective than in sexual reproduction (Latzel et al., 2016). However, few studies describing epigenetic inheritance in non-model plants have been published. In the next topic, we describe studies showing the role of epigenetic regulation in adaptation of non-model plants and some of this analysis highlighted the roles of putative epigenetic inheritance.

## Epigenetic Regulation in Adaptation of Non-Model Plants

A number of techniques have been used to identify epigenetic changes in plants, mainly DNA methylation profiling, which is the most studied epigenetic mechanism (Kurdyukov and Bullock, 2016). Recently, a high-resolution method for quantification of DNA methylation was development, the bsRADseq, which combines restriction site associated DNA sequencing with bisulfite sequencing (Trucchi et al., 2016). The technique of bissulfite sequencing, in which genomic DNA is treated with bisulfite to convert unmethylated cytosines to uracil, is usefull to obtain detail of genes methylation sequences, mainly in model plants (Cokus et al., 2008). However, studies of natural plant population have used mainly the Methyl-Sensitive Amplified Polymorphism (MSAP) approach (Box 1). MSAP is a technique that allows analyses of epigenetic variation for a high number of individuals (Schulz et al., 2013). In plants, MSAP was first used for identification of patterns of cytosine methylation in rice (Xiong et al., 1999). Given that epigenetic marks can result in changes of plants′ phenotypes, it is important to compare the variation in DNA methylation occuring between different plants in a population. Another method developed for epigenetic studies is a epiGBS, a reduced representation bisulfite method for exploration and comparative analysis of DNA methylation and genetic variation in hundreds of samples *de novo*, which can facilitate the study of plants that no have reference genome available (van Gurp et al., 2016). Here, we describe some studies that showed the variation in epigenetic marks in non-model plants (Table 1).

**BOX 1. Methyl-Sensitive Amplified Polymorphism – MSAP.**

Methyl-Sensitive Amplified Polymorphism (also referred as MS-AFLP) technique is a modification of the amplified fragment length polymorphism method (AFLP) based on the differential sensitivity of isoschizomeric restriction enzymes to site-specific cytosine methylation (Herrera and Bazaga, 2010). Thus, MSAP uses the same rare cutter *EcoRI* substituting the frequent cutter *MseI* by two enzymes that differ in their sensitivity to the methylation state of their recognition site 5′-CCGG, like *MspI* and HpaII (Schulz et al., 2013). For instance, MeCpG sites are recognized by *MspI* only, because *Msp*I does not cut when the inner cytosine is methylated and HemiMeCpCpG sites are recognized by *HpaII* only, because *Hpa*II does not cut when either or both cytosines are fully methylated or hemi-methylated (Schrey et al., 2013). On the other hand, sites hypermethylated and fully methylated are not cut by either enzyme and sites that are free from methlylation are recognized by both (Paun et al., 2010). Among the many benefits of using this technique, we highlighted the fact that this technique is a cost-effective allowing research on non-model systems including those that lack sequenced genomes. However, there are some shortcomings in this technique. One shortcoming is that MSAP cannot specify the region or gene influenced by methylation (Schrey et al., 2013). More recently, the Methylation Sensitive Amplification Polymorphism Sequencing (MSAP-Seq) approach was developed to allow the global sequence-based identification of changes in DNA methylation (Chwialkowska et al., 2017). MSAP-Seq has been validated in *Hordeum vulgare*, and can be used for DNA methylation analysis in crop plants with large and complex genomes and also non-model plants. In relation of technical short-comings of the MSAP technique, a problem is when both *Msp*I and *Hpa*II may fail to cut – in CHG and CHH methylation contexts, some methylated states can be missed (Schrey et al., 2013).

**Table 1:** Summary of studies with epigenetic in non-model plants.

Plant	Epigenetic modification	Environmental parameter	Heritable	Reference
*Viola cazorlensis*	DNA methylation	–	–	Herrera and Bazaga, 2010
Dactylorhiza species	DNA methylation	Water available in combination with temperature	–	Paun et al., 2010
*Laguncularia racemosa*	DNA methylation	Salt	Yes	Lira-Medeiros et al., 2010
*Alternanthera philoxeroides*	DNA methylation	Water available	–	Gao et al., 2010
*Elaeis guineensis*	DNA methylation	–	–	Ong-Abdullah et al., 2015
*Eucalyptus nitens*	DNA methylation	–	Yes	Thumma et al., 2009
*Pinus pinea*	DNA methylation	–	–	Saéz-Laguna et al., 2014
*Ilex aquifolium*	DNA methylation	Herbivory	–	Herrera and Bazaga, 2013
*Taraxacum officinale*	DNA methylation	Low nutrients, salt stress, JA application, SA application	Yes	Verhoeven et al., 2010


One of the earlier studies using MSAP was performed to examine the epigenetic differences between populations of the southern Spanish violet *Viola cazorlensis* (Herrera and Bazaga, 2010). Interestingly, the same samples used in this study were previously used in other analysis of variation in DNA sequence using AFLP methods (Herrera and Bazaga, 2008). Based on this, it was possible to correlate the genetic and epigenetic variation in *V. cazorlensis* population and methylation-based epigenetic differentiation of populations was associated with adaptive genetic divergence. Thus, the authors highlighted the importance of epigenetic modifications, and consequent phenotypic variation, in adaptation and evolution of natural and non-model population of plants. Analysis in three allotetraploid sibling orchid species, that differ radically in their geographic and ecological context, showed that ecological divergence of Dactylorhiza species is mostly due the epigenetic factors regulating gene expression in response to environmental stimulus (Paun et al., 2010). *D. traunsteineri*, *D. ebudensis*, and *D. majalis* showed species-specific epigenetic patterns that impacted the ecology, distribution, and evolution of these lineages through generations. Curiously, *D. majalis*, the species living in the most diverse environment showed less epigenetic variation than *D. traunsteineri*. However, authors indicate that the epigenetic constitution of an individual or species is sensitive to its environment, and water available in combination with temperature appears to be a key factor causing environmental allopatry in Dactylorhiza. In other words, the environmental conditions, mainly related to water availability and temperature, can result in changes of DNA methylation profiles, resulting in modification of phenotypic evolution and adaptation of plant population.

Genome-wide methylation profiling using MSAP revealed DNA methylation polymorphisms within and between natural populations. A study with two populations of the mangrove plant *Laguncularia racemose* grown in adjacent areas, but with different regimen of exposure to salt water, was performed using MSAP analysis to assess epigenetic variation in CpG methylation (Lira-Medeiros et al., 2010). This study was showed that the mangrove plants living near a salt marsh (SM) were hypomethylated (14.6% of loci had methylated samples) in comparison to the plants that live along a riverside (RS) (32.1% of loci had methylated samples). Is important to mention that those mangrove species can occur naturally in contrasting habitats and have different phenotype characteristics, for example, SM plants are small and have smaller leaf size compared to the RS plants. In addition, SM also had less epigenetic diversity than RS. Thus, CpG-methylation changes may be associated with environmental heterogeneity suggesting that epigenetic variation in natural plant populations is dependent of different environments. Interesting, AFLP analyzes of the same populations showed very little DNA variation, reinforcing the role of epigenetic variation in their adaptation. Analysis of DNA methylation profile of an invasive weed *Alternanthera philoxeroides* (alligator weed) also showed interpopulation difference in global DNA methylation in field plants (Gao et al., 2010). MSAP analysis revealed distinct DNA methylation patterns between aquatic and terrestrial plants, suggesting the potential of environmental factors to affect the methylation profile. Interestingly, 78.7% of epigenetic variation was observed within populations in response to different habitats. Despites this, 13.4 and 7.9% of epigenetic variation was also observed among geographic sites and between habitats within sites, respectively.

A study addressing phenotypic variation of native *Pinus pinea* plants showed a remarkable degree of phenotypic plasticity, despite having low levels of genetic variation. However, analysis of different vegetatively propagated trees showed a high degree of DNA methylation, suggesting the role of cytosine methylation in the improvement of *P. pinea* fitness under different environmental conditions (Saéz-Laguna et al., 2014). More recently, a study with the oil palm *Elaeis guineensis* revealed the impact of DNA methylation in an important characteristic of the fruit (Ong-Abdullah et al., 2015). Approximately, 75% of hypomethylated loci were transposons and repeats, while less frequent hypermethylated loci included genic sequences regions. This study showed that methylation near the Karma transposon predicts normal fruit and hypomethylation predicts homeotic transformation, parthenocarpy and marked loss of yield. Remarkably, the loss of Karma transposon methylation contributes to the origin of mantled plants, which is a somaclonal variant arising from tissue culture that drastically reduces yield, and has largely halted efforts to clone elite hybrids for oil production. In the tree *Eucalyptus nitens*, methylation of a CpG site in a gene involved in cellulose deposition is heritable, and the methylation pattern in DNA from either xylem or leaf tissues was similar, suggesting that methylation of this site is not tissue specific (Thumma et al., 2009).

Studies revealed that biotic stresses can also trigger an increase of the overall level of genomic methylation. Curiously, the methylation levels of some pathogen response or resistance genes are reduced (Peng and Zhang, 2009). This last profile results in up-regulation of genes involved in fast response to stress, but the increase in genomic DNA methylation may lead to a repression of the transcriptome. Application of jasmonic acid and salicylic acid is often used to experimentally mimic biotic attack and to induce defense pathways. Treatments with those phytohormones in the genetically identical apomictic dandelion (*Taraxacum officinale*) plants promote an increase in methylation changes in each of the treatments when compared with the control group. In addition, the epigenetic marks are largely heritable in the first generation (Verhoeven et al., 2010). In *Ilex aquifolium* (Aquifoliaceae) a link between herbivory, phenotypic plasticity and epigenetic changes was observed (Herrera and Bazaga, 2013). Some plants have leaves prickly and non-prickly, and the presence of this characteristic is a plastic defense response induced by mammalian browsing, which may reduce herbivory (Obeso, 1997). Herrera and Bazaga (2013) used MSAP to analyze the difference in DNA methylation in a heterophyllous tree producing two contrasting leaf, prickly, and non-prickly. Within heterophyllous branchlets, MSAP marker presence was significantly higher for prickly (mean ± SE = 0.681 ± 0.072) than for non-prickly (0.632 ± 0.077) leaves. The genome of prickly leaves was more demethylated in comparison of non-prickly leaf on the same branchlet. Interestingly, the plants that have these two putative leaves can be considered an epigenetic mosaic. Based on knowledge that epigenetic marks are transgenerationally heritable in plants the authors suggest that epigenetic mosaics can be translate into epigenetically heterogeneous progeny.

## Conclusion

Although part of the plants’ adaptation can be explained by the preexisting genetic variation in the populations, recent studies have shown that new stable phenotypes can be generated through epigenetic modifications in a few generations, contributing to the stability and survival of the plants in their natural habitat. The epigenetic regulation can cause dynamic changes, such as the plant hypersensitivity reaction (HR), changes in the structure of chromatin and influence the plant phenotype, contributing to the adaptation of native plants to stress. Thus, the knowledge of epigenetic contributions in phenotypic plasticity and hereditable variation is important to understand how natural population can adapt in different environmental condition, especially in a world context of climate change. Nevertheless, this is an area of study that clearly asks for additional investigation and the engagement of young scientists.

## Author Contributions

FT collected and analyzed the data and wrote the manuscript. AH commented and reviewed the article. PF critically reviewed the article and finished the manuscript.

## Conflict of Interest Statement

The authors declare that the research was conducted in the absence of any commercial or financial relationships that could be construed as a potential conflict of interest.
